# The potential harms of sedentary behaviour on cardiometabolic health are mitigated in highly active adults: a compositional data analysis

**DOI:** 10.1186/s44167-023-00015-7

**Published:** 2023-03-02

**Authors:** Wouter M. A. Franssen, Jarne Jermei, Hans H. C. M. Savelberg, Bert O. Eijnde

**Affiliations:** 1 REVAL-Rehabilitation Research Center, Faculty of Rehabilitation Sciences, Hasselt University, 3500 Hasselt, Belgium; 2 SMRC-Sports Medicine Research Center, BIOMED-Biomedical Research Institute, Faculty of Medicine and Life Sciences, Hasselt University, Agoralaan, Building A, Diepenbeek, 3500 Hasselt, Belgium; 3 Department of Nutrition and Movement Sciences, NUTRIM, School for Nutrition and Translation Research Maastricht, Faculty of Health, Medicine and Life Sciences, Maastricht University, 6229 Maastricht, The Netherlands; 4 Division of Sport Science, Faculty of Medicine and Health Sciences, Stellenbosch University, Stellenbosch, South Africa

**Keywords:** Cardiorespiratory fitness, Insulin sensitivity, Physical activity, Sitting time

## Abstract

**Background:**

Insufficient physical activity and sedentary behaviour (SB) are important factors that determine cardiometabolic health and the development of non-communicable diseases. The aim of this study was to investigate the modifying effects of moderate-to-vigorous physical activity (MVPA) on the association between SB and cardiometabolic health within highly active adults.

**Methods:**

In a cross-sectional design, 61 (male/female: 41/20) highly trained adults (age: 33.6 ± 10.7 years; BMI: 22.4 ± 2.3 kg/m^2^) performed a maximal cardiopulmonary exercise test from which indicators for peak performance were determined. Physical activity and SB were assessed using the activPAL3™ accelerometer. In addition, anthropometrics, blood pressure, plasma lipids and insulin sensitivity were assessed. These cross-sectional associations between a daily movement behaviour composition and cardiometabolic health parameters were investigated using a compositional data analysis approach.

**Results:**

Participants spent 600 ± 86 min/day in SB and engaged in almost 1.5 h per day of MVPA. No association was found between SB and cardiometabolic health related variables, whereas MVPA (β = 8.07 ± 2.18; r^2^ = 0.544; *p* < 0.001) was only significantly associated with oxygen uptake, relative to all other remaining behaviours.

**Conclusion:**

No associations were found between the time spent in SB and cardiometabolic health related outcomes, possibly due to the high amount of time spent in MVPA within highly active adults.

*Trial registration*: The present study was registered on the 14th of January 2022 at clinicaltrials.gov (NCT04711928).

**Supplementary Information:**

The online version contains supplementary material available at 10.1186/s44167-023-00015-7.

## Introduction

Insufficient physical activity is one of the major risk factors for the development of non-communicable diseases (NCD) and has been identified as the fourth leading cause of death worldwide [1]. Insufficient physical activity is defined as not reaching the recommended levels of 150–300 min per week spending in moderate-to-vigorous physical activity (MVPA), as stated by the 2020 World Health Organization guidelines [2]. Next to the time recommended to be spent in MVPA, it appears that sedentary behaviour also is an important factor that determines cardiometabolic health, NCD development and all-cause mortality [3–5]. Here, sedentary behaviour is defined as ‘any waking behaviour characterized by a low energy expenditure (≤ 1.5 metabolic equivalents), while being in a sitting or reclining posture’ [6]. In fact, during the past decade, emerging evidence clearly disclosed that prolonged sedentary behaviour is an interdependent contributor to cardiometabolic diseases and all-cause mortality, even in the presence of regular MVPA [7, 8]. However, several studies have observed that the association between sedentary behaviour and all-cause mortality could be modified, depending on the duration of time spent in MVPA [4]. Interestingly, large cohort studies suggested that spending more than 60 min per day in MVPA could beneficially modify (attenuate or even eliminate) the associations between time spent in sedentary behaviour with all-cause and cardiovascular disease mortality [4]. However, conclusions were only based on: (1) a large variety of time spent in MVPA (30–75 min per day); (2) the association between sedentary behaviour and all-cause mortality and (3) regression analyses of individual physical activity behaviours instead of analysing the combined effect of allocating time to different behaviours using compositional data analysis (CoDa) [4, 9–11]. Here, CoDa is combined with regression analyses using log-ratios rather than raw units. The large variation of time spent in MVPA necessary to mitigate the association between sedentary behaviour and all-cause mortality partly originates from the fact that these studies relied on self-reported data, differences in health status and fitness level of the participants and the use of hip or wrist worn accelerometers to assess MVPA and sedentary behaviour. It is well known that these measurement tools are prone to misclassification and, therefore, more reliable and valid instruments such as thigh-worn accelerometers are recommended [12].

Furthermore, although the modifying effects of MVPA on the association between sedentary time and all-cause mortality has been partly investigated, cardiometabolic health is a precursor of and strongly related to the development of NCDs [13]. Therefore, it is also important to know if the association between sedentary time and cardiometabolic health related outcomes can be modified by spending time in high amounts of MVPA. In addition, the majority of studies that examined the association between various movement behaviours (sleep, sedentary behaviour, standing, light-intensity physical activity [LPA] and MVPA) and cardiometabolic health have been performed in isolation by regression analyses, without adjustment for time spent in all other behaviours [4]. Given the finite nature of each day, time spent in one behaviour necessarily affects the time that remains to be spent in at least one other behaviour. Therefore, time spent in sleep, SB, standing, LPA and MVPA are related in a co-dependent manner. To date, new approaches as compositional data analysis have been recommended as a methodological analysis that accounts for this compositional approach [14] and has already been used within the field of sedentary behaviour and physical activity research [15]. Previous studies already showed that, within the composition, the proportions of time spent in SB showed statistically significant associations with body mass index [16], systolic and diastolic blood pressure [17], triglyceride concentration, high density lipoprotein cholesterol (HDL-cholesterol), C-reactive protein, plasma glucose and cardiometabolic risk score [18–20]. In addition, Janssen et al*.* performed a systematic review and found most consistent results for the relative contribution of MVPA to cardiometabolic health, whereas the relative contribution of sleep and LPA was controversial between studies [21].

To investigate if high amounts of time spent in MVPA are able to beneficially modify the inverse association between sedentary behaviour and cardiometabolic health, recreational athletes would be a suitable study population as their time spent in MVPA is corresponding to the highest physically active group from large cohort studies examining the modifying effects of MVPA on sedentary behaviour and all-cause mortality [4, 11]. Although it has been shown that recreational athletes have a better cardiometabolic health compared to the general population, the influence of sedentary behaviour is still unknown [22, 23].

Therefore, the aim of this study was to investigate the modifying effects of MVPA on the association between sedentary behaviour and cardiometabolic health using accurate accelerometer-derived measures within highly active adults. Here, a compositional data analyses approach will be used to account for all other movement behaviours such as sleep, standing time and LPA.

## Material and methods

### Subjects

Sixty competitive and recreational athletes aged between 18 and 65 years were locally recruited using online and paper advertisements. Subjects exercised ≥ 4 h per week [24] and different sport types were included based on the classification of Mitchell et al. and were restricted to dynamic MVPA as cycling, soccer and running [25].

Exclusion criteria were pregnancy, any known contradiction for physical activity, systolic blood pressure > 160 mm Hg, diastolic blood pressure > 100 mm Hg, more than 20 alcohol consumptions per week or subjects diagnosed with any known chronic disease or participants with contraindications for cardiopulmonary exercise testing (based on screening visit by general practitioner). All participants were informed in detail and were asked to provide written informed consent. The study was approved by the medical ethical committee of Hasselt University and performed at Hasselt University (Diepenbeek, Belgium) between March 2021 and June 2021 in accordance with the principles of the Declaration of Helsinki. The present study was registered on the 14th of January 2022 at clinicaltrials.gov (NCT04711928).

### Study design

The study was carried out according to an observational cross-sectional design. During a one-day study visit to Hasselt University the assessment of physical fitness and cardiometabolic health related outcomes including fasting blood samples, an oral glucose tolerance test, anthropometrics and body composition was performed. In addition, 24-h movement behaviours including sleep, sedentary behaviour standing time, and physical activity (LPA and MVPA) were measured. Then, associations were examined between sedentary behaviour, MVPA and these cardiometabolic health outcomes, relative to all other remaining behaviours.

### Study procedure

#### Screening

Following inclusion, participants were screened by their own general practitioner. This was based on a medical examination consisting of medical history and medication use. In addition, cardiovascular status was screened using a resting 12-lead electrocardiogram and resting blood pressure measurement.

#### Testing day

After a positive advice of the general practitioner, eligible participants were included for observational measurements during a testing day. Participants were instructed to refrain from strenuous physical exercise two days before the test day. Moreover, one day prior to each test day participants were requested not to consume alcohol. From midnight prior to examination, all subject refrained from consuming food, with the exception of water ad libitum to prevent changes on biochemical analysis and exercise physiology. First, in fasted state (at least twelve hours after the last meal) anthropometry and body composition using dual energy X‐ray absorptiometry were assessed and venous blood samples were collected. Subsequently, assessment of blood pressure and resting heart rate were performed. Next, an oral glucose tolerance test (OGTT) was performed to assess insulin sensitivity and beta cell function. After a light meal, cardiopulmonary exercise testing (CPET) was performed. Following all measurements, physical activity, standing time and sedentary behaviour were assessed using accelerometry (activPAL3™, PAL Technologies Ltd, Glasgow, Scotland) for seven consecutive days.

### Measurements

#### Physical activity and sedentary behaviour

Physical activity and body postures were quantified using the activPAL3™ activity monitor (PAL Technologies Ltd, Glasgow, UK). The device was enclosed with a nitrile sleeve and attached to the anterior mid-thigh of the participants right leg using an adhesive dressing (Tegaderm, 3 M, Minnesota, USA). Participants were instructed to wear the device for a period of 7 consecutive days and 24 h per day, without removing it at any time. The inclinometer function of the activPAL™ was used to accurately assess time spent in sleeping, sedentary behaviours (sitting or lying) and standing, while the accelerometer function was used to examine physical activity including step count and step cadence (low intensity physical activity [< 100 steps/min] and MVPA [> 100 steps/min]) [26]. Furthermore, short (< 30 min) and prolonged (> 60 min) sedentary bouts were identified. All variables were determined from the ActivPAL™ recordings using proprietary software (PALanalysis V8, PAL Technologies Ltd, Glasgow, UK). Data from the ActivPAL software were also processed using customised software written in MATLAB R2013b (MathWorks, Natick, MA, USA) to automatically determine sleeping times [27]. It has been shown that this is a valid and accurate algorithm to determine wake and bedtimes on an individual level, which was highly associated with self-reported wake and bed times [27].

#### Anthropometry and body composition

Body height was measured to the nearest 0.1 cm using a wall-mounted Harpenden stadiometer, with participants barefoot. Body weight (in underwear) was determined using a digital-balanced weighting scale to the nearest 0.1 kg. BMI was calculated from weight and height measurements (weight/height^2^). Waist and hip circumferences were measured to the nearest 0.1 cm using a flexible metric measuring tape with participants barefoot (in light clothes) in standing position. Waist circumference was measured at the midpoint between the lower rib margin and the top of the iliac crest. Hip circumference was measured at the widest circumference of the hip at the level of the greater trochanter. Both measures were assessed in triplicate and the mean value of the triplicate measurements was used in the analysis. Waist-to-hip ratio was calculated by dividing waist circumference (cm) by hip circumference (cm). Whole body fat, lean tissue mass and bone mineral density were evaluated using dual energy X-ray absorptiometry (Hologic Series Delphi-A Fan Beam X-ray Bone Densitometer, Vilvoorde, Belgium).

#### Insulin sensitivity, beta cell function and plasma lipids

After antecubital catheter placement, fasting blood samples were obtained for the measurement of cardiometabolic risk factors. Serum separation and sodium fluoride (NaF) containing BD vacutainer™ tubes (Becton, Dickinson and Company, Franklin lakes, NY, USA) were collected. To obtain plasma, NaF tubes were immediately centrifuged at 1300×*g* for 15 min. Serum tubes coagulated for at least 30 min prior to centrifuging at 1300×*g* for 15 min. All centrifugation steps were performed at room temperature (21 °C). Supernatants were immediately portioned into aliquots and frozen at − 20 °C and subsequently moved to a − 80 °C freezer until analysis. Fasting glucose, insulin, total cholesterol, HDL-cholesterol, low density lipoprotein cholesterol (LDL-cholesterol) and triglyceride concentrations were automatically assessed on the Roche Cobas 8000 (Roche Diagnostics International Ltd, Rotkreuz, Switzerland).

A standard 5-point oral glucose tolerance test (OGTT) was performed for assessment of whole body/tissue specific insulin sensitivity and beta cell function. Subjects ingested a solution (250 ml) containing 75 g dextrose, and venous blood samples were obtained at t = 0, 30, 60, 90 and 120 min for assessment of venous glucose and insulin concentration. From glucose and insulin concentrations, whole-body and tissue specific insulin resistance and beta cell function was estimated (Additional file 1).

#### Blood pressure

After an initial resting period of 20 min with participants in a seated position in a quiet room with constant temperature (21 °C), blood pressure (BP) was measured at least 3 times at 2-min intervals until blood pressure was stabilized using an electronic sphygmomanometer (Omron®, Omron Healthcare, IL, USA) from the left arm and documented as the mean value of the 3 final measurements. Mean arterial pressure (MAP) was calculated as MAP = systolic BP + (2 × diastolic BP) / 3.

#### Clustered cardiometabolic risk score

A clustered cardiometabolic risk score (CCMR) was computed with the aid of the following variables: waist circumference, triglycerides, HDL-cholesterol, MAP and fasting plasma glucose [28, 29]. The sex specific standardized value of each individual variable was calculated as follows: z-score = [individual value – sample mean]/ standard deviation (SD). Subsequently, HDL-cholesterol z-scores were inverted and z-scores of all variables were averaged to form a CCMR.

#### Cardiorespiratory fitness

A cardiopulmonary exercise test was performed up to volitional exhaustion using an electronically braked cycle ergometer (eBike Basic®, General Electric GmbH, Bitz, Germany), controlled by the Metamax (Metalyzer II® 3B Cortex, Leipzig, Germany) [30]. After a 5-min warm-up phase, an incremental exercise cycling period with an initial workload of 80W for male and 40W for female, and increasing workload of 40W per minute for male and 30W per minute for female was performed. During incremental exercise a cycling frequency of 60–70 revolutions per minute (rpm) had to be maintained. The test was ended when the participant failed to maintain a pedal frequency of at least 60 rpm. With the aid of continuous pulmonary gas exchange analysis oxygen uptake (V̇O_2_), carbon dioxide output (V̇CO_2_) and the respiratory gas exchange ratio (RER) was collected breath-by-breath and averaged every ten seconds. Heart rate (HR) was continuously monitored and averaged every ten seconds using the H10 Polar heart rate monitor (Polar Electro Oy, Kempele, Finland). All participants were verbally encouraged during exercise testing to achieve maximal effort, based on RER, maximal HR and blood lactate levels (satisfaction of 2/3 of the following criteria: RER > 1.15; maximal HR ≥ predicted -10 beats per minute; post exercise lactate level > 8.0 mmol/L).

#### Data analyses

Statistical analysis was performed by IBM SPSS® version 27.0 (IBM SPSS Statistics for Windows, Chicago, IL, USA). Data were expressed as mean ± SD. A Shapiro–Wilk test was used to test normality of the data (*p* < 0.05). Three different groups were created according to tertiles of total sedentary time (SB_low_, SB_intermediate_ and SB_high_) to compare cardiometabolic health related outcomes between different amounts of sedentary behaviour. Large cohort and cross-sectional studies already showed that people spend on average between 8.5 and 10 h per day [4, 9–11, 31]. In addition, in these cohort studies sedentary time ranged from 8 to 13 h per day. Here, the dose–response relations between sedentary time and mortality increased slightly from about 7.5 to 9 h per day and were more pronounced at greater than 9.5 h per day. Based on these results, three different groups were created with an average sedentary time of 8.4, 10.0 and 11.6 h per day, which exactly fits within the range of these large cohort trials. Comparisons between groups (low, intermediate and high sedentary time) were tested using the Fischer’s exact test for categorical variables. For continuous variables a one-way ANOVA (Bonferroni post-hoc comparison test) was used for normally distributed data and the Kruskal–Wallis test (Dunn’s post-hoc comparison test) for abnormally distributed data.

Variables of sleep, physical activity (standing, LPA and MVPA) and sedentary time were analysed using compositional data analyses (CoDa) approach with the aid of the R package Shiny (Shiny V.1.0.5, RStudio, Boston, USA, 2017), as described before [32]. The CoDa approach is based on the fact that if the time spent in one behaviour is changed, it will inevitably affect the time in at least one other behaviour within that day. Data with this inherent dependency in a way that they add up to a constant sum are constrained or compositional [33].

Therefore, the composition of the day was defined as the proportions (p) of time spent in five different moment behaviours: sleeping, sedentary behaviour, standing, LPA and MVPA (the method is described in detail by Chastin et al. [32]). Here, the compositional mean of all individuals was calculated by normalizing the geometric means of all individual components (sleeping, sedentary behaviour, standing, LPA and MVPA) in such a way they add up to 1 (adjusted for the total day time of 1440 min). Then, the overall geometric (g_overall_) mean for each component was calculated by combining all participants:

1 $${g}_{overall}=1 \left(24h\right)={p}_{sleeping}+{p}_{sedentary \, behaviour}+{p}_{standing}+{p}_{LPA}+{p}_{MVPA}$$

In addition, the geometric mean was calculated for each component within the subgroups with a low, intermediate and high sedentary time (g_SBlow_, g_SBintermediate_ and g_SBhigh;_ g_subgroup_). To characterize the movement behaviours of the individual subgroups relative to the geometric mean of the overall composition the log-ratio of the geometric mean within a group and the overall geometric mean of the individual components was calculated as:

2 $$\mathrm{Relative \, difference \, between \, subgroups}:log\left(\frac{{g}_{subgroup}}{{g}_{overall}}\right)$$

The variability within the data was described as the variability of each behaviour relative to the variability of all other behaviours using a variation matrix. Here, a log-ratio variance close to zero indicated high co-dependence (proportionality) between the behaviours. Here, a value close to zero implies that the two components involved in the ratio are highly proportional (high co-dependence). To treat and interpret the data correctly, information that contains parts of a composition needs to be expressed relative to the other parts as log ratios [33]. Therefore, time spent in sleep, sedentary behaviour, standing, LPA and MVPA were transformed into isometric log ratio (ILR) coordinates given by the Eqs. 3 − 6:

3 $$\mathrm{ILR}/\mathrm{ln}(\mathrm{SB}:\mathrm{ other \, behaviours}) = \sqrt{\frac{4}{5} }\mathrm{ln }\left(\frac{\mathrm{SB}}{\sqrt[4]{\mathrm{Sleep}\cdot \mathrm{Standing}\cdot \mathrm{LPA}\cdot \mathrm{MVPA}}}\right)$$

4 $$\mathrm{ILR}/\mathrm{ln}(\mathrm{standing}:\mathrm{ other \, behaviours})=\sqrt{\frac{4}{5}} \mathrm{ ln }\left(\frac{\mathrm{Standing}}{\sqrt[4]{\mathrm{Sleep}\cdot \mathrm{SB}\cdot \mathrm{LPA}\cdot \mathrm{MVPA}}}\right)$$

5 $$\mathrm{ILR}/\mathrm{ln}(\mathrm{LPA}:\mathrm{ other \, behaviours})=\sqrt{\frac{4}{5}} \mathrm{ ln }\left(\frac{\mathrm{LPA}}{\sqrt[4]{\mathrm{Sleep}\cdot \mathrm{SB}\cdot \mathrm{Standing}\cdot \mathrm{MVPA}}}\right)$$

6 $$\mathrm{ILR}/\mathrm{ln}(\mathrm{MVPA}:\mathrm{ other \, behaviours})=\sqrt{\frac{4}{5}} \mathrm{ ln }\left(\frac{\mathrm{MVPA}}{\sqrt[4]{\mathrm{Sleep}\cdot \mathrm{SB}\cdot \mathrm{Standing}\cdot \mathrm{LPA}}}\right)$$

Thus, these ILRs express the ratio of sedentary behaviour, standing, LPA or MVPA to time in all other behaviours.

Multivariate linear regression analyses were applied to examine the association between the daily composition of time spent in sleep, sedentary behaviour, standing, LPA and MVPA as independent variables with cardiometabolic health related outcomes. Models were also adjusted for potential confounders including sex, age, smoking status, chronic disease and medication. Correction for multiple testing was implemented using the Benjamini–Hochberg false discovery rate (FDR) method, with FDR < 0.05 considered as statistically significant [34]. A *p*-value < 0.05 (2-tailed) was considered statistically significant. The sample size calculation was performed using GPower v. 3.1 (Düsseldorf, Germany). Madden et al. have shown a significant association (effect size f^2^: 0.23) between the cardiometabolic risk score and the isometric log-ratio transformation for time spent sedentary, independent of age and biological sex in elderly [18]. Based on a statistical power > 0.8 and a two-sided alpha of 0.05 it was calculated that a sample size of 53 individuals had to be included in the present study. Taking into account a drop-out rate of 10%, the number of participants to include in this study was at least 58.

## Results

### Subject characteristics and cardiometabolic health related outcomes

**Fig. 1:**
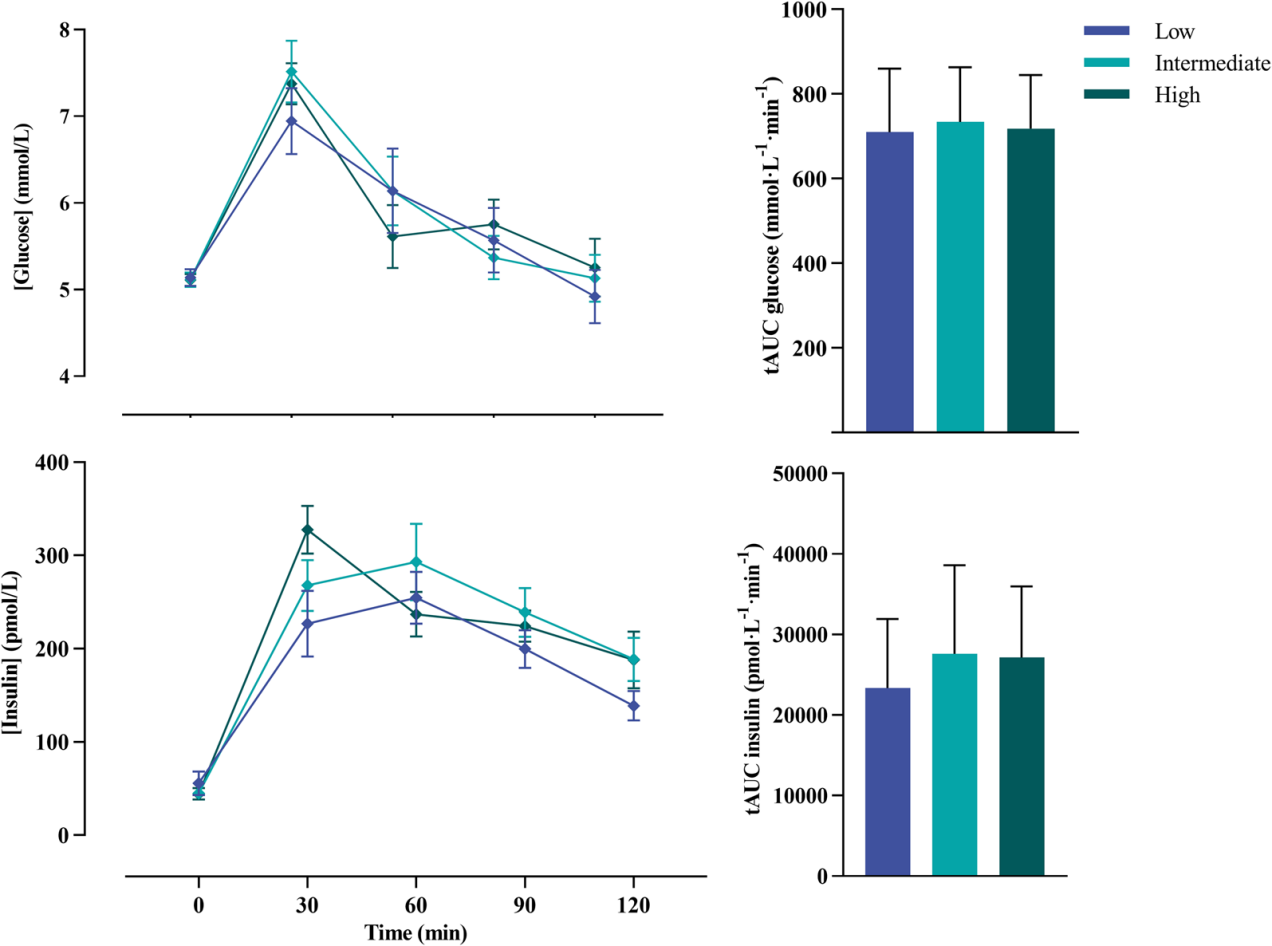
Glucose and insulin concentrations during a 2-h oral glucose tolerance test (left hand panel) and the average area under the curve (right hand panel) of the three different groups (low, intermediate and high sedentary time). Data are presented as mean ± standard error of the mean. *tAUC*  total area under the curve

A total of 72 participants were screened for study entry of which 61 individuals effectively participated in the study. Exclusion was due to a spending less than 4 h per week on structured MVPA (n = 7) and age restrictions (n = 4). Participants had a mean age of 33.6 ± 10.7 years (range: 18.9–64.5 years of age), a BMI of 22.4 ± 2.3 kg/m^2^ (range: 17.2–31.0 kg/m^2^) and a maximal oxygen uptake of 53.5 ± 10.0 mL kg^−1^ min^−1^ (range: 38.6–79.7 mL kg^−1^ min^−1^). Furthermore, the total population consisted of 41 males (67%) and 20 females (33%). All participants wore the ActivPAL for a period of 7 consecutive days and 24 h hours per day and spent 452 ± 39 min/day (31%) sleeping, 600 ± 86 min/day (42%) in sedentary behaviours, 219 ± 59 min/day (15%) standing, 83 ± 29 min/day (6%) in LPA and 87 ± 51 min/day (6%) in MVPA. No significant between group differences (low, intermediate and high sedentary time) were found for anthropometrics, body composition, blood pressure (Table 1) and cardiometabolic health outcomes (Table 2 and Fig. 1) between groups.

**Table 1:** Subject characteristics

General features	Low (n = 20)	Intermediate (n = 20)	High (n = 21)	*p*-value
Age (years)	38.4 ± 8.2	32.1 ± 12.9	34.5 ± 9.4	0.085
Sex (m/f)	14/6	13/6	13/7	0.499
Body weight (kg)	72.6 ± 12.2	69.8 ± 10.4	69.5 ± 10.8	0.627
Body height (cm)	177.3 ± 9.0	177.7 ± 8.1	175.9 ± 8.5	0.764
BMI (kg/m^2^)	23.0 ± 2.9	22.0 ± 2.1	22.3 ± 2.0	0.397
Waist circumference (cm)	80.4 ± 10.0	77.8 ± 8.5	76.4 ± 6.9	0.327
Hip circumference (cm)	89.8 ± 6.0	87.7 ± 6.6	86.7 ± 6.7	0.306
Waist-to-hip-ratio	0.89 ± 0.07	0.89 ± 0.05	0.88 ± 0.05	0.822
Lean mass (kg)	56.0 ± 6.1	54.1 ± 8.9	54.2 ± 9.8	0.783
Fat mass (kg)	12.5 ± 5.5	11.7 ± 3.6	11.5 ± 4.0	0.733
Fat mass (%)	17.6 ± 6.9	17.2 ± 5.0	17.0 ± 6.0	0.949
Weekly sport hours	9.2 ± 3.6	8.2 ± 2.9	7.9 ± 3.0	0.430
Maximal oxygen uptake (mL min^−1^)	3991 ± 987	3755 ± 869	3619 ± 896	0.429
Maximal oxygen uptake (mL kg^−1^ min^−1^)	55.0 ± 11.1	53.7 ± 9.5	51.9 ± 9.5	0.610
Sleeping time (min/day)	438 ± 35	468 ± 32	448 ± 45	0.057
Sedentary time (min/day)	501 ± 34	600 ± 24 ^a^	695 ± 37 ^b, c^	** < 0.001**
Standing time (min/day)	267 ± 46	214 ± 48^a^	179 ± 48^c^	** < 0.001**
LPA (min/day)	109 ± 23	81 ± 23^a^	60 ± 19^b, c^	** < 0.001**
MVPA (min/day)	126 ± 66	77 ± 33^a^	59 ± 19^c^	** < 0.001**
Step count	13,225 ± 3815	9904 ± 2694^a^	7714 ± 2563^c^	** < 0.001**
Sitting bouts < 30 min	378 ± 83	376 ± 96	397 ± 134	0.780
Sitting bouts 30–60 min	116 ± 39	150 ± 45	167 ± 48^c^	**0.002**
Sitting bouts > 60 min	76 ± 44	137 ± 61	243 ± 129^b, c^	** < 0.001**
Smoking status (n)				0.638
Current	1	0	0	
Former	3	2	2	
Never	16	18	19	
Chronic disease (n)				0.242
Respiratory	1	0	3	
Cardiovascular	0	1	0	
Medication (n)				0.242
ACE inhibitor	0	1	0	
Bronchodilator	1	0	3	

*p* < 0.05 are shown in bold

Data are expressed as mean ± SD. *ACE*  angiotensin-converting enzyme, *BMI*  body mass index, *LPA*  light intensity physical activity, *MVPA*  moderate-to-vigorous physical activity

^a^*p* < 0.05 low vs. intermediate

^b^*p* < 0.05 intermediate vs. high

^c^*p* < 0.05 low vs. high

**Table 2:** Cardiometabolic risk factors between groups with low, intermediate and high sedentary time

	Low (n = 20)	Intermediate (n = 20)	High (n = 21)	*p*-value
*Cardiovascular health*				
Systolic blood pressure (mm Hg)	118 ± 10	120 ± 14	118 ± 10	0.818
Diastolic blood pressure (mm Hg)	71 ± 9	73 ± 7	71 ± 7	0.553
Mean arterial pressure (mm Hg)	87 ± 9	89 ± 8	87 ± 7	0.600
Resting heart rate (bpm)	62 ± 6	61 ± 7	59 ± 9	0.129
Total cholesterol (mmol/L)	4.27 ± 0.65	4.22 ± 0.77	4.05 ± 0.82	0.619
HDL cholesterol (mmol/L)	1.61 ± 0.47	1.47 ± 0.28	1.62 ± 0.43	0.402
LDL-cholesterol (mmol/L)	2.30 ± 0.46	2.34 ± 0.75	2.04 ± 0.67	0.446
Triglycerides (mmol/L)	0.75 ± 0.22	0.89 ± 0.27	0.81 ± 0.24	0.190
CCMR	− 0.03 ± 0.63	0.12 ± 0.78	− 0.09 ± 0.41	0.547
*Glucose tolerance*				
Fasting glucose (mmol/L)	5.2 ± 0.4	5.1 ± 0.4	5.1 ± 0.3	0.963
Fasting insulin (pmol/L)	56 ± 57	45 ± 20	44 ± 28	0.574
Glucose 120 min (mmol/L)	4.9 ± 1.4	5.1 ± 1.2	5.3 ± 1.5	0.738
Insulin 120 min (pmol/L)^a^	139 ± 70	189 ± 101	188 ± 139	0.511
Matsuda index	8.16 ± 2.89	8.42 ± 3.90	8.47 ± 3.21	0.955
IGI^a^	90 ± 112	80 ± 122	142 ± 76	0.103
HOMA-IR^a^	1.44 ± 0.51	1.49 ± 0.77	1.47 ± 0.98	0.979
HOMA-B (%)^a^	101.3 ± 101.9	80.7 ± 29.5	77.9 ± 42.2	0.468
mISI	0.37 ± 0.24	0.29 ± 0.15	0.33 ± 0.19	0.476
HIRI	21.8 ± 8.2	24.8 ± 6.6	27.0 ± 5.2	0.056

Data are expressed as mean ± SD

*BP*  blood pressure, *bpm*  beats per minute, *CCMR*  clustered cardiometabolic risk score, *HDL*  high-density lipoprotein, *HIRI* hepatic insulin resistance index, *HOMA-B*  homeostatic model assessment of β-cell function, *HOMA-IR*  homeostatic model assessment of insulin resistance, *HOMA-S*  homeostatic model assessment of insulin sensitivity, *IGI*  insulinogenic index, *LDL*  low-density lipoprotein, *mISI*  muscle insulin sensitivity index

*p < 0.05. ^a^Differences between groups were assessed using the Kruskal–Wallis test due to the abnormal distribution of the data

### Physical activity and sedentary behaviour

Time spent in sedentary bouts of more than 60 min (low: 76 ± 44 vs. high: 243 ± 129; *p* < 0.001) and 30–60 min (low: 116 ± 39 vs. high: 167 ± 48; *p* = 0.002) was significantly lower in the low sedentary behaviour group compared to the high sedentary behaviour group. Although there was no significant difference between groups with regard to sleeping time (p = 0.057), the difference in sedentary time was due to significant differences in standing time (low: 267 ± 46 min/day vs. intermediate: 214 ± 48 min/day; *p* = 0.003 and vs. high: 179 ± 48;* p* < 0.001) and physical activity of all intensities including, LPA (low: 109 ± 23 min/day vs. intermediate: 81 ± 21 min/day; *p* < 0.001 and vs. high: 60 ± 19;* p* < 0.001) and MVPA (low: 126 ± 66 min/day vs. intermediate: 77 ± 33 min/day; *p* = 0.002 and vs. high: 59 ± 19;* p* < 0.001), which were significantly higher in the low sedentary behaviour group compared to the intermediate and the high sedentary behaviour group (*p* < 0.001). These higher volumes of physical activity were also reflected by a significantly higher step count in the low sedentary behaviour group, compared to the intermediate (*p* = 0.003) and high sedentary behaviour group (*p* < 0.001).

**Fig. 2:**
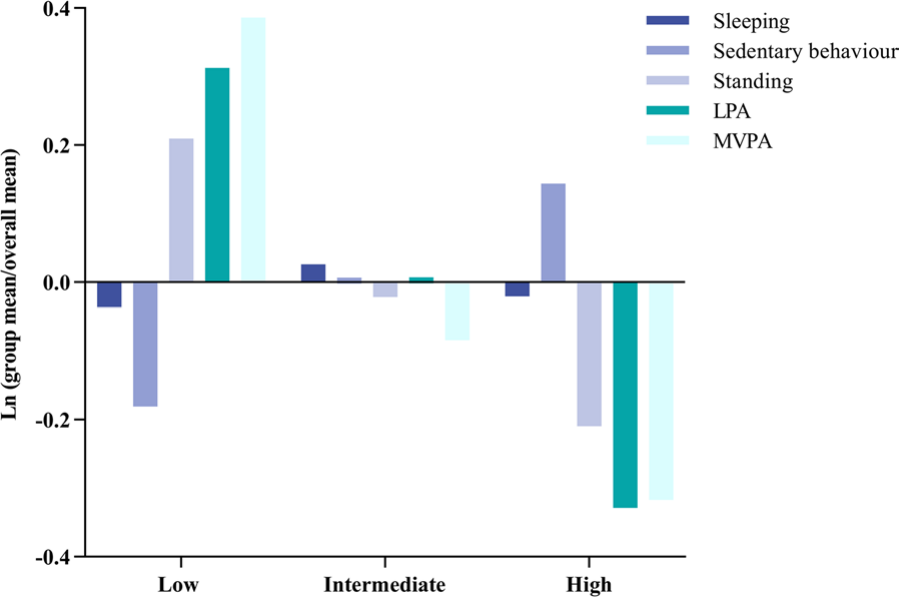
Compositional analysis of the relative importance of the group (low, intermediate or high) mean time spent in sleep, sedentary behaviour, standing, LPA and MVPA with respect to the overall mean time composition. *LPA*  light intensity physical activity, *MVPA*  moderate-to-vigorous physical activity

The analysis of the daily composition of behaviour categories showed that in the low sedentary behaviour group the proportion of time spent in both standing (21%), LPA (32%) and MVPA (38%) was higher compared to the overall mean composition, whereas the high sedentary behaviour group spent less time in standing (− 21%), LPA (− 32%) and MVPA (− 31%) levels relative to the entire sample (Fig. 2). The variation matrix showed the highest log-ratio for sedentary time/MVPA (0.398), reflecting low co-dependence between these behaviours (Table 3). The lowest values were found for the log-ratio of sleeping time in relation to sedentary time (0.025), standing time (0.109) and LPA (0.130), reflecting high co-dependence between sleeping and these behaviours.

**Table 3:** Variation matrix of time spent in sleep, sedentary time, standing time, LPA and MVPA

	Sleeping time	Sedentary time	Standing time	LPA	MVPA
Sleeping time	0	0.025	0.109	0.130	0.236
Sedentary time	0.025	0	0.207	0.312	0.398
Standing time	0.109	0.207	0	0.048	0.170
LPA	0.130	0.312	0.048	0	0.329
MVPA	0.236	0.398	0.170	0.329	0

Variances close to zero implies a high co-dependence (proportionality) between variables

*LPA*  light-intensity physical activity, *MVPA*  moderate-to-vigorous intensity physical activity

### Associations between physical activity, sedentary behaviour and cardiometabolic health

A higher sedentary time was associated with a higher HIRI (β = 8.78 ± 3.17; r^2^ = 0.150; *p* = 0.008), whereas a negative association was found with the maximal oxygen uptake (β = − 9.19 ± 3.50; r^2^ = 0.492; *p* = 0.011), relative to all other remaining behaviours (Table 4). After correcting for multiple testing, no association remained statistically significant. Spending more time in MVPA was associated with a higher maximal oxygen uptake (β = 8.07 ± 2.18; r^2^ = 0.544; *p* < 0.001) and a lower insulin concentration after 120 min of the OGTT (β = − 60.27 ± 29.04; r^2^ = 0.327; *p* = 0.043), HIRI (β = − 4.71 ± 2.13; r^2^ = 0.110; *p* = 0.031) and triglyceride concentration (β = − 121 ± 0.073; r^2^ = 0.128; *p* = 0.019), relative to all other remaining behaviours. After adjustments for multiple testing, only the association between MVPA and oxygen uptake remained statistically significant. No associations between sleeping time, standing time or LPA and markers of cardiometabolic health were found, relative to all other remaining behaviours. Analyses with the average daily physical activity composition were also performed, but no statistically significant associations were found with cardiometabolic health variables.

**Table 4:** Multiple linear regression analyses of the relationship between isometric log-ratio (ilr) coordinates of sedentary time and MVPA and cardiometabolic health related outcomes

	ILR/ln(sedentary time: other behaviours)				ILR/ln(MVPA: other behaviours)			
	r^2^	B	SE	p-value	r^2^	B	SE	p-value
Waist circumference^a^	0.450	− 0.015	0.017	0.373	0.447	− 0.008	0.011	0.455
Systolic blood pressure	0.452	4.663	4.004	0.249	0.444	− 1.814	2.646	0.496
Diastolic blood pressure	0.318	4.252	2.654	0.313	0.282	− 3.161	2.144	0.146
Fat mass percentage^a^	0.499	0.062	0.055	0.262	0.492	− 0.025	0.036	0.481
Oxygen uptake per kg	0.492	− 9.191	3.504	**0.011**	0.544	8.074	2.178	** < 0.001**
Fasting glucose	0.160	0.024	0.167	0.885	0.164	− 0.057	0.109	0.603
Glucose 120 min	0.185	0.806	0.603	0.187	0.206	− 0.707	0.394	0.078
Fasting insulin	0.033	− 10.592	18.37	0.567	0.028	1.579	12.078	0.896
Insulin 120 min	0.314	79.04	44.32	0.080	0.327	− 60.27	29.04	**0.043**
Matsuda index	0.151	− 0.641	1.621	0.694	0.150	0.351	1.023	0.733
IGI	0.094	240.6	111.4	0.422	0.081	− 144.3	73.55	0.055
HOMA-IR	0.142	− 0.031	0.100	0.756	0.141	0.006	0.065	0.931
HOMA-B	0.150	− 0.103	0.096	0.288	0.139	0.041	0.062	0.510
mISI	0.155	− 0.112	0.091	0.227	0.135	0.034	0.062	0.583
HIRI	0.150	8.775	3.173	**0.008**	0.110	− 4.708	2.128	**0.031**
tAUC insulin	0.222	0.054	0.014	0.134	0.201	− 0.085	0.044	0.061
tAUC glucose	0.157	63.58	60.46	0.298	0.149	− 31.45	40.18	0.437
Total cholesterol	0.321	0.396	0.301	0.194	0.322	− 0.268	0.197	0.180
Triglycerides	0.058	0.136	0.116	0.247	0.128	− 0.177	0.073	**0.019**
HDL-cholesterol^a^	0.273	0.062	0.046	0.186	0.273	0.04	0.03	0.182
LDL-cholesterol	0.278	0.105	0.267	0.697	0.320	− 0.317	0.17	0.068
CCMS	0.374	0.099	0.228	0.667	0.412	− 0.311	0.154	0.094

All models were adjusted for sex, age, smoking status, chronic disease and medication. *B*  unstandardised beta coefficients, *HIRI*  hepatic insulin resistance index, *HDL*  high-density lipoprotein, *HOMA-B*  homeostatic model assessment of β-cell function, *HOMA-IR*  homeostatic model assessment of insulin resistance, *ILR*  isometric log-ratio, *IGI*  insulinogenic index, *kg*  kilogram, *LDL*  low-density lipoprotein, *mISI*  muscle insulin sensitivity index, *MVPA*  moderate-to-vigorous physical activity, *SE*  standard error, *tAUC*  total area under curve, *CCMS*  clustered cardiometabolic risk score. Significant associations are shown in bold

^a^Variables were log-transformed due to the abnormal distribution of the data

## Discussion

In the current study, we aimed to investigate the modifying effects of MVPA on the association between sedentary behaviour and cardiometabolic health in highly active adults. Participants included in this study were highly active, by engaging in almost 1.5 h per day of MVPA. This far exceeds the 30 min per day as recommended by the current physical activity guidelines. However, despite these high levels of MVPA, they engaged in different amounts of sedentary behaviours ranging from 7 to 13 h a day. We found that the cardiometabolic health risks attributed to these high amounts of sedentary time could fully be mitigated by the high levels of MVPA and possibly by high levels of cardiorespiratory fitness (CRF). This was confirmed by multivariate linear regression analyses showing that the CRF, reflected by the maximal oxygen uptake, was positively associated with MVPA.

Despite the well-established health benefits of daily MVPA, increasing evidence suggests that sleep, SB and LPA also have important consequences for (cardiometabolic) health [35]. Although it has been shown that spending 9 h per day in sedentary behaviours is associated with the risk of all-cause mortality [4], in the present study no significant differences in cardiometabolic health related outcomes were found between the low (126 min of MVPA), intermediate (77 min of MVPA) and high sedentary (60 min of MVPA) groups. This suggests that 60 min of MVPA per day (high sedentary behaviour group) mitigates the detrimental effects of prolonged sedentary behaviour (11.5 h/day, of which 4 h were spent in bouts > 60 min). This is similar to a previous meta-analysis of Ekelund et al. who found that between 60 and 75 min per day of leisure time physical activity of moderate intensity was necessary to eliminate the risk of mortality associated with sedentary behaviour [10]. However, their results were based on self-reported data. Other harmonized meta-analyses based on accelerometer measured physical activity found that 30–40 min of MVPA on a daily basis were enough to attenuate the risk of all-cause mortality [9, 11]. However, Chastin et al. showed that at high sedentary time (> 11 h) the benefits of these lower MVPA levels, compared to the 60 min of our study, might have been completely attenuated [9].

In contrast, intervention studies have found that 60 min of daily physical exercise could not compensate the negative effects of sedentary behaviour on cardiometabolic health when the rest of the day was spent in sitting pursuits [36, 37]. From this, it could be suggested that the beneficial effects from exercise are fully blunted due to physical inactivity, a phenomenon termed “exercise resistance” [37]. However, in comparison with our study population in which participants already performed exercise for at least the past 2 years, the conclusions of Duvivier and Coyle were based on relatively short-term interventions (4 days). In addition, their 60-min training was performed at 65% of maximal heart rate, which means training at moderate physical activity intensity. In our study it was only possible to measure MVPA instead of moderate and vigorous physical activity (VPA) as separate intensities. Therefore, no conclusions could be made based on the contribution of these intensities as a stand-alone factor. Nevertheless, it has been shown that athletes spent more time in VPA compared to the normal population [38], and therefore, it could be assumed that in the current study the proportion of time spent in VPA was higher than the population studied by Duvivier et al*.* Here, it is possible that physical activity at higher intensities can counteract the effects of prolonged sedentary behaviour. Recent research has proposed that the proportion of time spent in VPA, compared to moderate intensity, might be more important for reducing the (cardiovascular disease) mortality risk [39]. Therefore, replacing sedentary behaviour with physical activity of higher intensities on the longer-term could be an important contributor to the beneficial effects of MVPA on cardiometabolic health within these highly trained athletes. Indeed, it has already been shown that a higher contribution of VPA to total physical activity levels is associated with additional health effects [40]. Although VPA is time-efficient, structured exercise-based sessions at this intensity are not feasible most of the time. Interestingly, Stamatakis et al. proposed a new paradigm that allows VPA to be more accessible with the aid of the regular accumulation of vigorous intermittent lifestyle physical activity (VILPA) as part of daily living, such as carrying shopping bags and stair climbing [41]. They recently showed that 3 bouts per day (4.4 min per day) was associated with a reduction in all-cause, CVD and cancer mortality, whereby non-exercisers appeared to elicit beneficial health effects of similar magnitude to exercisers [42]. This emphasizes the potential of promoting VILPA next to other physical activity intensities during leisure time.

No differences on cardiometabolic health related outcomes were found between groups, which was also confirmed by the multivariate regression analyses. This is in contrast with Zheng et al*.* who found that total sedentary time and prolonged sedentary bouts were positively associated with several cardiometabolic biomarkers within highly active young males [43]. However, the associations should be interpreted with caution since multiple linear regression analyses have performed, which may lead to the results being coincidental, as shown in the current study. Furthermore, no associations were found between cardiometabolic health, standing time and LPA which indicates that the time spent in these behaviours is far less important when a certain threshold of MVPA is reached.

MVPA was positively associated with maximal oxygen uptake, relative to all other remaining behaviours. Although it has been shown that there is a strong interrelationship between sedentary time, MVPA and CRF, these factors might have an independent association with cardiometabolic health [44]. Indeed, Van der Velde et al. showed that even individuals with a higher CRF may be at increased risk for metabolic diseases due to prolonged sitting and that both sedentary time and CRF were independently associated with the metabolic syndrome and type 2 diabetes mellitus [45]. However, this study was performed in the general population, in which parameters of CRF were lower compared to athletic populations as included in the current study. Interestingly, it has already been shown that CRF may modify the association between sedentary behaviour and cardiometabolic health [46]. Nauman et al*.* found that high levels of CRF compensated the deleterious health consequences related to engaging in sedentary behaviour for > 7 h per day [46]. Indeed, in the current study, despite the fact that we found equal high CRF levels across the groups engaging in different levels of sedentary behaviour, no differences were found in cardiometabolic health related outcomes. This suggests that sedentary behaviour may be a less important determinant of cardiometabolic health in persons with adequate CRF. It has been shown that high levels of CRF fully eliminated the detrimental effects of sedentary behaviour, even when they did not meet the current PA recommendations of 150 − 300 min per week engaging in MVPA [46, 47]. Therefore, it could be suggested that people with a high CRF may provide favourable effects against the deleterious consequences of prolonged sitting. This might also explain why no associations between LPA, standing and markers of cardiometabolic health were found. Indeed, McCarthy et al*.* demonstrated less metabolic benefit from LPA breaks in individuals with a CRF (± 58 mL kg^−1^ min^−1^) comparable to that in the present study [48]. This supports the concept that individuals with higher CRF gain less pronounced health benefits from reducing sedentary behaviour [48].

Although, to our knowledge, this was the first study that investigated associations between sedentary behaviour and cardiometabolic health with CoDa within highly active adults, several limitations could be addressed in future research. Firstly, the cross-sectional nature of our study limited the ability to infer causality. Prospective longitudinal studies are highly recommended to investigate the direct association between these variables. Secondly, although the ActivPAL™ is the gold standard for measuring sedentary behaviour [12], discriminating between moderate and vigorous intensity physical activity is not possible. In this population, it is assumed that VPA was more performed compared to MPA, which could explain why no more associations were found between sedentary time and cardiometabolic health. To further unravel the direct associations between moderate and vigorous physical activity and cardiometabolic health, measurement tools which can perfectly distinguish between these movement behaviours are warranted. Third, food intake was not considered in this study, a factor that may also relate to cardiometabolic health and the development of NCDs [49]. Fourth, due to small sample size the conclusion should be interpreted with caution. In addition, although we corrected for sex in our statistical analyses, it is warranted in future research to discriminate between males and females to better understand sex differences across the various movement behaviours.

## Conclusion

Taken together, it can be concluded that, despite the high levels of sedentary behaviour, high levels of MVPA are likely to mitigate the inverse association between sedentary behaviour and cardiometabolic health. It seems that engaging in at least 60 min of MVPA may be viable to protect the potential harms of prolonged sitting.

## Supplementary Information

**Additional file 1. Calculations of insulin sensitivity and beta cell function parameters.**

## Data Availability

The datasets used and/or analysed during the current study are stored in a permanent repository and are available from the corresponding author on reasonable request.
