# Supplementary material for: The potential harms of sedentary behaviour on cardiometabolic health are mitigated in highly active adults: a compositional data analysis
Source: J Act Sedentary Sleep Behav. 2023 Mar 2;2:6. doi: 10.1186/s44167-023-00015-7 (PMC11960216; doi:10.1186/s44167-023-00015-7)
Supplement: Supplementary file 1 — Additional file 1. Calculations of insulin sensitivity and beta cell function parameters. [file 44167_2023_15_MOESM1_ESM.docx]

**Additional file 1**

**Calculations insulin sensitivity and beta cell function**

From glucose and insulin concentrations whole-body insulin resistance was estimated using the homeostatic model assessment for insulin resistance (HOMA-IR) and the Matsuda index. The HOMA-IR was calculated by: fasting glucose (mmol/L) * fasting insulin (µU/mL) / 22.5 [1], and the Matsuda index was calculated as: 10,000/√[fasting glucose (mg/dL) * fasting insulin (µU/mL)) * (mean glucose during OGTT (mg/dL) * mean insulin during OGTT (µU/mL)] [2]. Beta cell function was estimated by calculation of the insulinogenic index (IGI): ratio of increment of insulin (µU/mL) and glucose (mg/dL) in the first 30 min of OGTT and the HOMA-B: 20 * fasting insulin (µU/mL) / (fasting glucose [mmol/L] − 3.5) [1]. Tissue specific insulin resistance was calculate using the hepatic insulin resistance index (HIRI) and the muscle insulin resistance index (mISI). The HIRI was calculated as the product of the tAUCs for glucose and insulin during the first 30 min of the OGTT (glucose 0 – 30 [tAUC in mg/dL h] * insulin 0 – 30 [tAUC in µU/mL h]) and the mISI was calculated as the rate of decay of glucose concentration during the OGTT divided by the mean insulin concentration during the OGTT in mg/dL/min/ µU/mL)*.* The rate of decay was calculated as the slope of the least square fit to the decline in glucose concentration from peak to nadir, as described by Vogelzangs *et al* [3].The total area under the curve (tAUC) for glucose and insulin for the 2 hour period was calculated using the trapezoidal rule [4].

1. Singh, B. and A. Saxena, *Surrogate markers of insulin resistance: A review.* World J Diabetes, 2010. **1**(2): p. 36-47.

2. Matsuda, M. and R.A. DeFronzo, *Insulin sensitivity indices obtained from oral glucose tolerance testing: comparison with the euglycemic insulin clamp.* Diabetes Care, 1999. **22**(9): p. 1462-70.

3. Vogelzangs, N., et al., *Metabolic profiling of tissue-specific insulin resistance in human obesity: results from the Diogenes study and the Maastricht Study.* Int J Obes (Lond), 2020. **44**(6): p. 1376-1386.

4. Matthews, J.N., et al., *Analysis of serial measurements in medical research.* Bmj, 1990. **300**(6719): p. 230-5.
